# The Effect of Velvet Antler Proteins on Cardiac Microvascular Endothelial Cells Challenged with Ischemia-Hypoxia

**DOI:** 10.3389/fphar.2017.00601

**Published:** 2017-09-04

**Authors:** Xiang Xiao, Shuqiang Xu, Lin Li, Min Mao, Jinping Wang, Yanjun Li, Ziwei Wang, Fei Ye, Li Huang

**Affiliations:** ^1^National Integrated Traditional and Western Medicine Center for Cardiovascular Disease, China-Japan Friendship Hospital Beijing, China; ^2^Emergency Office, National Health and Family Planning Commission Beijing, China; ^3^Department of Pharmaceutical, China-Japan Friendship Hospital Beijing, China; ^4^Graduate School, Beijing University of Chinese Medicine Beijing, China; ^5^Institute of Materia, Chinese Academy of Medical Sciences and Peking Union Medical College Beijing, China

**Keywords:** Chinese medicine, velvet antler, vascular endothelial injury, ischemia-hypoxia, Akt

## Abstract

Velvet antler (VA) is a precious traditional Chinese medicine that is capable of repeated regeneration. Based on the Chinese medicine theory of coordination the heart and kidneys, VA has been employed to treat heart diseases, including ischemic heart disease, heart failure, and arrhythmia. We examined the effects of VA proteins on primary cardiac microvascular endothelial cells (CMECs) that were subjected to ischemia-hypoxia (IH) to investigate their effects on and mechanism of action in the treatment of ischemic heart disease. Velvet antler proteins (VA-pro) were extracted with water as the solvent, the ultrasonic wave method, and freeze-drying technology; then it was analyzed by Nano LC-MS/MS. In addition, the role of VA-pro in cell viability, proliferation, apoptosis, and mitochondrial membrane potential (MMP) were evaluated by the MTS assay, the EdU assay, the Annexin V-FITC/PI double-staining assay, and the JC-1 assay, respectively. Cell migration were evaluated by the scratch assay and the Transwell assay. The expression of apoptosis-associate proteins, Akt and p-Akt, and tube formation in Matrigel of CMECs were also detected. In total, 386 VA-pro were identified. Our results showed that IH significantly reduced the viability of the CMECs (*P* < 0.001) and suppressed copies of DNA to hold back CMEC proliferation (*P* < 0.001). The OD of control group was 1.81 ± 0.08 and IH group OD was 1.25 ± 0.03. After suffering with IH for 46 h, CMECs were 75% less likely to migrate (*P* < 0.001), and its tubule formation ability and MMP were also decreased (*P* < 0.001). VA-pro treatment resulted in an improvement in CMECs’ viability and proliferation (*P* < 0.001). Such as, the OD of 0.5, 1, and 2 mg/ml rose to 1.56 ± 0.5, 1.74 ± 0.1 and 1.65 ± 0.1, respectively. Similarly, CMECs’ migration (for the scratch assay *P* < 0.001, for the Transwell assay *P* < 0.05) and tubule formation (*P* < 0.05) ability were better after treated with VA-pro. At the same time, the stability of MMP was retained preferably (*P* < 0.001). 50% apoptosis was induced after CMECs were cultured in IH conditions (*P* < 0.001), while VA-pro decreased the number of apoptotic cells (*P* < 0.001). All above results showed that 1 mg/ml VA-pro produced maximum results. Furthermore, the expression of pro-apoptosis proteins was higher, but the expression of anti-apoptosis proteins was lower in the IH group (*P* < 0.05); VA-pro reversed these changes (*P* < 0.001). These findings suggest that VA-pro ameliorate CMEC injuries induced by IH via regulating the PI3K/Akt signaling pathway.

## Introduction

Velvet antler (VA, Cornu Cervi Pantotrichum) has been a precious traditional Chinese medicine (with the Chinese name of Lu Rong) for 2,000 years (Wu et al., 2013). It can strengthen the liver and kidneys and boost energy. Moreover, there is a close relationship between the heart and the kidneys in the Chinese medicine theory of heart and kidneys harmony, especially the warming and supplement function of kidney-*yang* to heart-*yang*. VA is used to treat heart diseases based on this theory. For example, VA is the basis of xin-shen pills in the Categorized Collection of Medical Formulas (*Yi Fang Lei Ju*), and VA and ginseng are paired herbs in cardiotonic and diuretic prescriptions. VA is also employed to treat arrhythmia (Liu et al., 2014), ischemic heart disease, and heart failure (Zheng, 2013); furthermore, additional effects of VA on these diseases and the mechanism of action are being researched (Shao et al., 2012), with the VA effects on vascular endothelial cells as a new point in related research (Chen et al., 2009; Wang and Shi, 2009).

Endothelial cell dysfunction is associated with the development of myocardial infarction, coronary disease, and other cardiovascular diseases so it is a key point in the clinical treatment of cardiovascular diseases. On the other hand, some studies (Park et al., 2004; Gao et al., 2010) on VA proteomics analyses found that the main active pharmacological ingredients of VA are proteins and polypeptides, which play important roles in the medicinal function of VA and they have an obvious advantage in new drug research (Zha et al., 2012). Our recent research found that VA proteins (VA-pro) could influence the migration and proliferation of endothelial progenitor cells (Xiao et al., 2017). Therefore, we wanted to elucidate whether VA-pro protects CMECs from ischemia-hypoxia (IH)-induced injury in this study.

## Materials and Methods

### Velvet Antler Tissue Preparation

Velvet antler tissue was prepared as previously described (Xiao et al., 2017). Fresh VA, produced in the Dongfeng Sika Deer Farm, Liaoyuan City, Jilin Province of China, was obtained from 4-year-old sika deer (*Cervus nippon* Temminck), which were at the early stage of their fast-growing antlers. The deer were bred in Jilin Province of China and identified by Professor Chunsheng Liu of the Department of Chinese Medicine Identification at the Institute of Traditional Chinese Medicine, Beijing University of Chinese Medicine. After administering general anesthesia, the antlers were removed by cutting the proximal region with a surgical hand saw and washed with 75% ethanol. Blood was then drawn from the VAs with a vacuum instrument (SHZ-IIIB, Tan Shi Vacuum Equipment Co., Ltd., Linhai, Zhejiang, China) for 6 h in 4°C, sliced, and freeze-dried by lyophilizer (FreeZone Plus 12 Liter Cascade Console Freeze Dry System, -84°C, LABCONCO, Kansas City, MO, United States). The temperature was -60°C and the baro-vacuum was 12 Pa until the VA quality change was less than 0.1 g. With superfine grinding technology, the freeze-dried VA was superfinely comminuted under the cryogenic environment and 54.08 g lyophilized VA powder was created. The dehydration percentage of VA was 67.02%. The powder was stockpiled in sealed tubes and numbered XX-DGF01 to XX-DGF04.

### Extraction of Velvet Antler Protein (VA-pro)

The method has been described briefly before (Xiao et al., 2017). Lyophilized VA powder was weighed and placed in a large beaker, mixed with ultrapure water at the proportion of 1:10, with vortex oscillation blending, then the lyophilized VA powder was mixed for 5 s and then paused for 15 s. Using homogenate and immersion methods, with 12 cycles in an ice-bath, the VA was ultrasonically extracted for 2 h. The supernate was obtained in a 50-ml centrifuge tube and the precipitate was removed by centrifugation (12,000 rpm, 30 min), the supernate was stored at -80°C for less than 12 h, and then freeze-dried by lyophilizer (FreeZone Plus 12 Liter Cascade Console Freeze Dry System, -84°C, LABCONCO, Kansas City, MO, United States) for 72 h. The precipitate was processed according to the above steps three times, and then all of the freeze-dried VA-pro were mixed, numbered from XX-VApro01 to XX-VApro03, and stored at -80°C. The protein content was measured using a bicinchoninic acid (BCA) protein assay kit (Thermo Fisher Scientific, Waltham, MA, United States). According to the specifications, the diluted bull serum albumin (BSA) standards and BCA working reagent (WR) were prepared first: 200 μl of WR and 25 μl of samples or 25 μl of diluted albumin (BSA) standards were added into the 96-well plate and mixed at 37°C for 30 min. The plate was removed and set at room temperature for 10 min, and the optical density (OD) was counted by microplate spectrophotometer (Spectra MR, Dynex, United States). The VA-pro mass concentration was 465.56 μg/mg.

### Nano LC-MS/MS Analysis

We digested 120 μg of VA-pro with 4 μg of trypsin for 24 h at 37°C and we added 1 μg of trypsin for another 12 h at 37°C, then the salt was removed with a C18 column (Phenomenex StrataTM, Torrance, CA, United States) and the desalted peptide mixture was obtained. Nano LC-MS/MS analysis was performed as previously described (Xiao et al., 2017).

### MS Data Analysis

After HPLC-MS/MS measurement, the raw data were imported into the PD (Proteome Discoverer 1.3, Thermo Fisher Scientific, Waltham, MA, United States) to be filtered. The collected data files were processed and sent to a MASCOT server (Version 2.3.0, Matrix Science) for peptide identification using the UniProt database^1^ with the Ruminantia taxonomy constraint (The retrieval parameters shown in **Table 1**).

**Table 1:** MASCOT retrieval parameters.

Parameter	Options
Mascot version	2.3.0
Fixed modification	Carbamidomethyl (C)
Variable modification	Oxidation (M), Gln→Pyro-Glu (N-term Q), iTRAQ 8 plex (K), iTRAQ 8 plex (Y), iTRAQ 8 plex (N-term)
Peptide tol.	15 ppm
MS/MS tol.	20 mmu
Max missed cleavages	1
Enzyme	Trypsin
Database	uni_Ruminantia-9845, Number of sequences:93869

The sample was annotated based on the GO database^2^ and the KEGG database^3^ .

**FIGURE 1:**
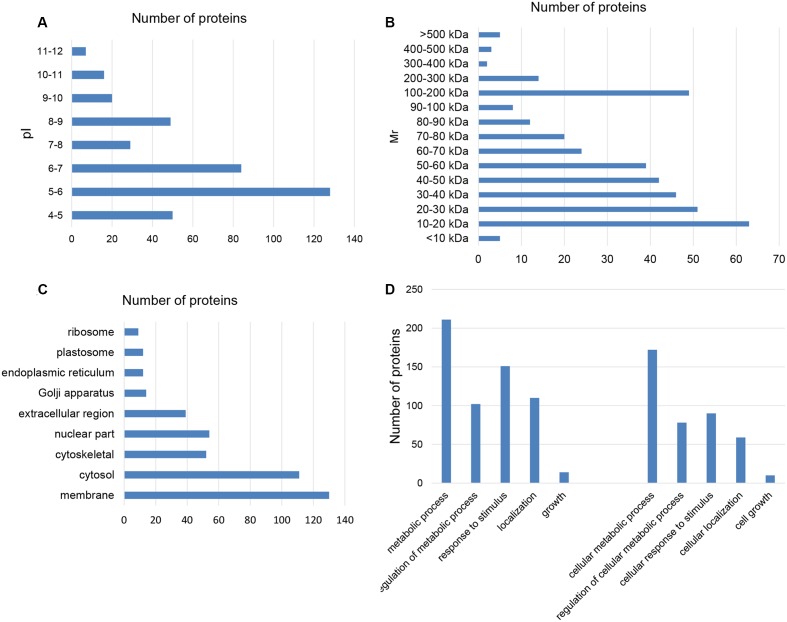
Nano LC-MS/MS analysis and function analysis. VA-pro pI **(A)** and MW **(B)** ranged from 4.14 to 11.57 and 6 to 800 kDa. The cellular component **(C)** mainly involved nine parts. In comparing the number of proteins between biological function and cell function **(D)**, the proportion was 81.5, 76.5, 59.6, 53.6, and 71.4%.

The total number of VA-pro identified by the MASCOT search was 386 using the taxonomy Ruminantia (Uniprot), so there were 145 uncharacterized proteins. The 386 pI ranged from 4.14 to 11.57, with a pI of 4.0 to 7.0 (68.4%) and 8.0 to 9.0 (12.8%) for most proteins (**Figure 1A**). The molecular weight (MW) ranged from 6 to 800 kDa, with most of the proteins from 10 to 80 kDa (74.4%) and 100 to 200 kDa (12.8%) (**Figure 1B**). For the VA-pro function analysis (GO annotation), part of cellular component results are shown in **Figure 1C**. As for the biological processes, 211 proteins were involved in the metabolic process, 102 proteins were involved in metabolic regulation, 151 proteins were involved in the biological process of response to stimulus, 110 proteins were involved in the localized biological processes, and 14 proteins were involved in the growth of related biological processes. In addition, there were 172 proteins involved in cell metabolism, 78 proteins involved in the regulation of cell metabolism, 90 proteins involved in cellular responses to stimuli, 59 proteins involved in cellular localization, and 10 proteins involved in the biological process of cell growth (**Figure 1D**). Further, 38 proteins were involved in the cell apoptosis process, 27 proteins were related to cell migration, 4 proteins were closely related to cell proliferation, and 2 proteins played a role in mitochondrial changes during apoptosis.

### Isolation and Culture of Cardiac Microvascular Endothelial Cells (CMECs)

The CMECs were cultured from 2-day-old SD rat pup hearts using mature and accepted methods. The protocol was approved by the Animal Care and Welfare Committee of the Clinical Medicine Institute in China-Japan Friendship Hospital. Rat pups’ hearts were excised, minced, and digested in 2% Collagenase II solution (Gibco, Grand Island, NE, United States) for 7 min at 37°C five times. The digested product was collected in a centrifuge tube containing serum and was filtered through a 70-m nylon cell strainer, then the cells were collected and adhered differentially. CMECs were cultured in Endothelial Cell Medium (ECM, Sciencell, Carlsbad, CA, United States), consisting of 500 ml of basal medium, 25 ml of fetal bovine serum (FBS), 5 ml of endothelial cell growth supplement (ECGS), and 5 ml of penicillin/streptomycin (P/S) solution in standard incubator conditions. CMECs were determined by the presence of vWF VIII (Abcam, Cambridge, United Kingdom) and PECAM-1/CD31 (Abcam, Cambridge, United Kingdom).

### Cell Treatment and Group

After two passages, CMECs were used for the experiment, treated with ECM for 24 h, then the control cells were cultured in complete ECM while other cells were washed with PBS twice, then cultured in FBS-free medium with different VA-pro concentrations. CMECs remained in culture at 37°C with hypoxia conditions (5% CO_2_-95%N_2_) for 46 h. The control cells were cultured in a regular incubator at 37°C, 5% CO_2_.

To find an effective dose range of VA-pro, we set up 6 dose groups (0.25, 0.5, 1, 2, 4, and 8 mg/ml, with no FBS in the medium) within the ischemia IH group (no FBS in the medium, hypoxia conditions) and the hypoxia group (H group, 10% FBS, hypoxia conditions). The control group of cells was cultured with complete medium at 37°C, 5% CO_2_.

### Cell Viability

Cell viability was determined using the Cell Titer 96^®^Aqueous One Solution Reagent (MTS assay, PROMEGA, United Kingdom). Cells were cultured in a 96-well plate. Before measuring, each well medium was replaced by 100 μl of new medium and we added 20 μl of reagent, then we cultured the cells for 3 h at 37°C, 5% CO_2_. A blank well with the same volume of medium but without cells was as a control well. The optical density (OD) was measured by microplate spectrophotometer (Spectra MR, Dynex, United States).

### Cell Proliferation

Cell proliferation was determined using an EdU kit (RIB BIO, Guangzhou, China), with blue and red indicating the Hoechst 33342- and EdU-labeled cells, respectively. After adding the EdU (20 μM), cells were cultured for 4 h. Then, cells were fixed with 4% (w/v) formaldehyde in PBS for 10 min, and other testing steps were performed in accordance with the operation manual. The results were detected by a high-intensity scanning system and analyzed by MetaXpress software (Molecular Devices, Sunnyvale, CA, United States).

### Tubule Formation Assay on Matrigel

The Matrigel was thawed (BD, San Jose, CA, United States) at 4°C for 3 h. We added 100 μl of Matrigel to each well of a 48-well plate and then left the Matrigel to solidify at 37°C for 30 min. With 3 × 10^4^ CMECs seeded on top of the Matrigel layer in 250 μl of culture medium, the cells were subsequently incubated at 37°C, 5% CO2. The tube network was observed by bright field microscope (Leica Microsystems, Germany) during a 12-h period. The branch point cells were calculated in 4 random fields per well. These images were analyzed using NIS Elements AR Analysis 4.10.00 (Nikon, Tokyo, Japan).

### Scratch Assay

We cultured 3 × 10^5^ CMECs in 12-well plates for 24 h. Subsequently, confluent cell monolayers were scratched with a P200 pipette tip to make three parallel “wounds” in each well, and we washed the cells with PBS two times. After cells were incubated with different treatments, the wounds were photographed and counted using a bright field microscope, and the pictures were analyzed with Prism 5.0 (GraphPad Inc., La Jolla, CA, United States).

### Transwell Assay

Invasion chambers (Corning, NY, United States) were also used to detect CMEC migration. We added 150 μl of cell FBS-free suspension (1 × 105 cells/ml) to the inserts and medium with different drug concentrations to the lower chamber. After the treatment, non-invasive cells in the upper chamber were removed with cotton swabs and invasive cells were stained with DAPI. We counted six random fields for each chamber (magnification, ×200) using a microscope (Leica Microsystems, Germany).

### Annexin V-Fluorescein Isothiocyanate (FITC)/Propidum Iodide (PI) Double-Staining Assay

To quantify cellular apoptosis in the early and late stages, cells were seeded in a 6-well plate, and after treatment, they were harvested and processed for the apoptosis assay using an Annexin V-FITC/PI kit (BD, San Jose, CA, United States) following the indicated protocol specified by the manufacturer. They were examined using an EPICS XL flow cytometer and EPICS XL ADC system (Beckman Coulter, Porterville, CA, United States). Data were analyzed using Cell Quest software (BD, San Jose, CA, United States).

### Mitochondrial Membrane Potential

The mitochondrial membrane potential (MMP) was measured using a JC-1 kit (5,5′,6,6′-tetrachloro-1,1′,3,30′-tetraethyl-imidacarbocyanine, BD, San Jose, CA, United States). We dissolved JC-1 in DMSO and diluted it with medium at 1:99 when it was used. We stained 1 × 10^6^ cells with 500 μl of working solution at 37°C for 15 min in the dark, washed with 1× assay buffer twice and resuspended in 1× assay buffer, followed by analysis using flow cytometry. A minimum of 10,000 events was acquired to assess JC-1 fluorescence in both FL-1 (monomers, green fluorescence) and FL-2 channels (aggregates, red fluorescence) using the EPICS XL flow cytometer.

### Western Blotting

To further explore the mechanisms involved in the effect of VA-pro on CMECs and the possible involvement of PI3K/Akt signaling, an inhibitor group was set and LY294002 (10 μM) was added to the medium. IH-injured cells were washed and lysed with RIPA lysis buffer (containing protease inhibitors and phosphatase inhibitors). With 20 μl/well loaded, the proteins were resolved on a 12% polyacrylamide gel and transferred to a nitrocellulose membrane. Membranes were blocked with 5% non-fat dry milk for 2 h, then incubated with primary antibodies against Akt (1:1000, CST), p-Akt (1:1000), Bcl-2, Bax, caspase-3, cleaved caspase-3 (all from CST, Boston, MA, United States), and GAPDH (1:1000, Abcam, Cambridge, United Kingdom) overnight at 4°C. Membranes were then washed and further incubated with goat anti-rabbit antibody (1:5000, LI-COR, Lincoln, NE, United States) at room temperature for 1 h. Bands were visualized and analyzed using Odyssey SA and Image Studio version 4.0 (LI-COR Biosciences, Lincoln, NE, United States).

### Immunofluorescence

For immunofluorescence, cells grown on glass cover slips were fixed with 4% paraformaldehyde at room temperature for 15 min. Cells were washed three times with PBS for 5 min and then they were treated with 0.3% triton-100, blocked with 3% BSA (PBS) for 1 h, and then were immunolabeled with primary antibody (anti-CD31 at 1:100, anti-vWF at 1:200) overnight at 4°C. They were then washed with PBS, and immunolabeled with Alexa 488-conjugated secondary antibody or Alexa 555-conjugated secondary antibody (1:200, all from CST, Boston, MA, United States) for 1 h at room temperature in the dark. Cells were washed in PBS, and the nuclei were stained with DAPI for 10 min. Glass cover slips were upended on the glass slide before storing at 4°C. The cells were imaged and counted using an inverted fluorescent microscope (Nikon, Tokyo, Japan) and Leica TCS SP5 confocal microscope (Leica, Germany). Digital images were prepared using NIS Elements AR Analysis 4.10.00 and Adobe Photoshop CS6 software (Adobe Systems, San Jose, CA, United States).

### Statistics

Quantitative data were presented as means ± SD and were analyzed by GraphPad Prism 5 (GraphPad Software, Inc., United States). Statistical differences among groups were assessed by ANOVA followed by Tukey’s or Dunnett’s posttest. *P* < 0.05 was considered to be significant. All of the experiments were set in three parallel groups in support of a three-time repeat.

## Results

### Identification of Cardiac Microvascular Endothelial Cells (CMECs)

**FIGURE 2:**
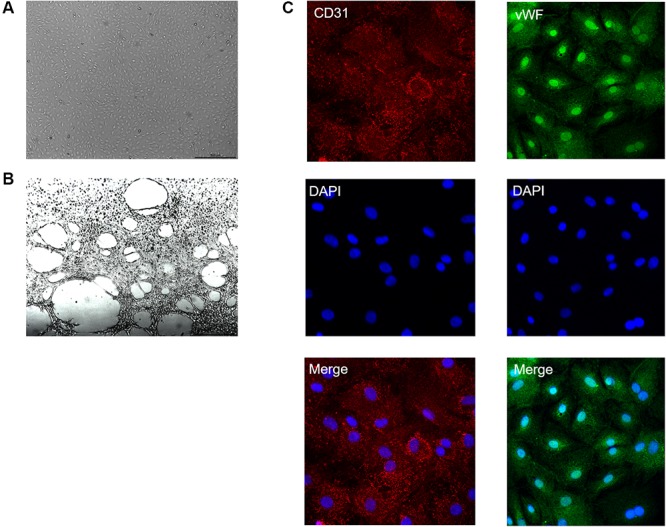
Identification of CMECs. **(A)** Microscopic photograph of CMECs. **(B)** CMEC tubule formation assay on Matrigel. **(C)** Markers of CMECs (CD31 and vWF) were positive with immunofluorescence staining. Photographs were taken by confocal microscope (magnification: ×630).

After two passages, CMECs were ovoid and could form tubes on the Matrigel (**Figures 2A,B**). Cells were identified by their surface markers vWF and CD31 (**Figure 2C**). The morphology, tubes, and the positive surface marker manifested a successful culture of CMECs.

### VA-pro Improved the Viability of CMECs Subjected to Ischemia-Hypoxia

**FIGURE 3:**
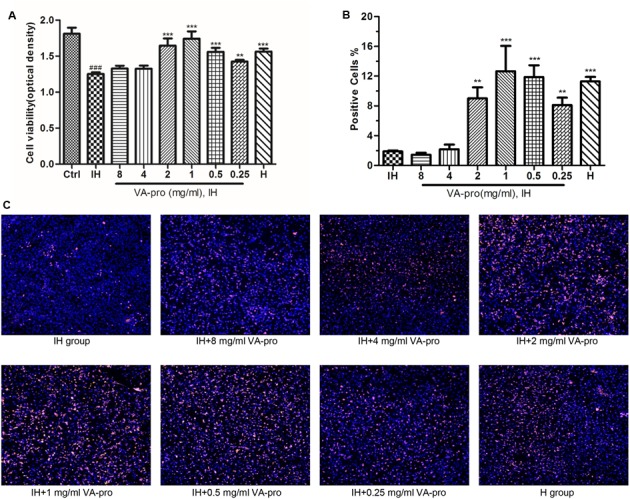
VA-pro improved IH-induced CMECs viability. The cell viability was evaluated by MTS assay and the OD (optical density) was measured by microplate spectrophotometer **(A)** (*n* = 5 per group). Cell proliferation was determined using an EdU kit and detected by a high intensity scanning system. Blue and red indicate Hoechst 33342- and EdU-labeled cells, respectively **(C)**. Data were obtained and analyzed by MetaXpress software **(B)** (*n* = 3 per group). ^###^*p* < 0.001 vs control group; ^∗∗^*p* < 0.01 and ^∗∗∗^*p* < 0.001 vs IH group.

Cardiac microvascular endothelial cell viability in the IH group was significantly reduced (*P* < 0.001). VA-pro treatment resulted in an improvement in CMEC viability, and the best dose was 1 mg/ml (*P* < 0.001) because the group of cells almost had the same viability as the control group. Other dose groups were no different when compared with the H group, except for 4 and 8 mg/ml (**Figure 3A**). As shown in **Figures 3B,C**, IH suppressed copies of DNA to hold back CMEC proliferation. VA-pro treatment resulted in better cell proliferation, especially at 0.5 and 1 mg/ml (*P* < 0.001). These resulted suggested that VA-pro improved the viability of CMECs subjected to IH.

### VA-pro Attenuated the Apoptosis of CMECs Subjected to Ischemia-Hypoxia

**FIGURE 4:**
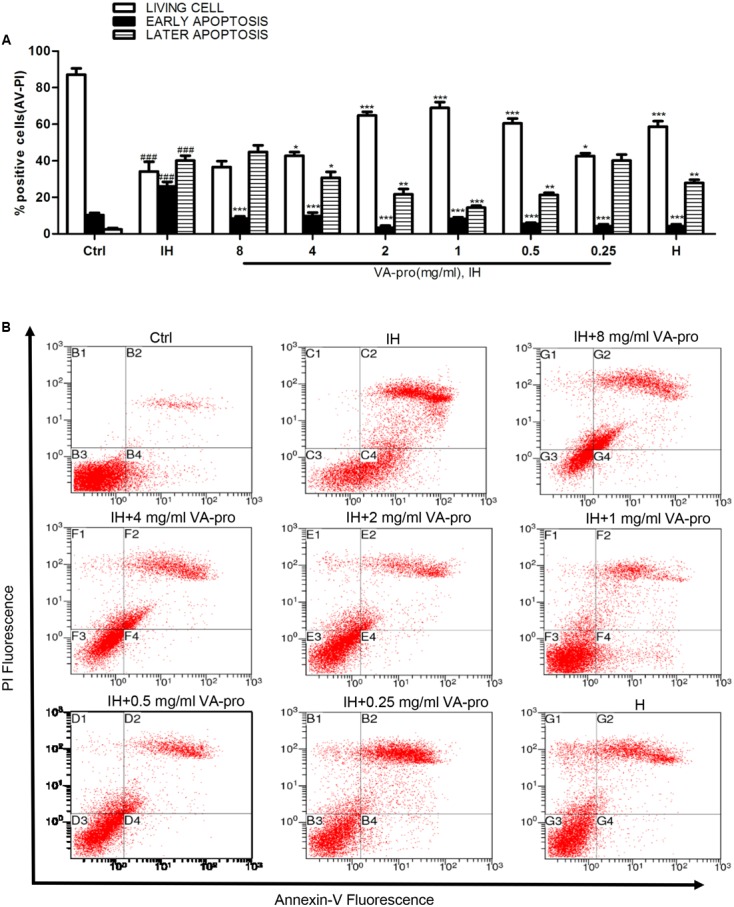
VA-pro attenuated CMEC apoptosis. Apoptosis was assessed with an Annexin V-FITC/PI kit and detected by flow cytometry (*n* = 3 per group). **(A,B)** Flow cytometric dot plot shows that AV^-^ PI^-^cells (normal cells) are in the third quadrant, AV^+^ PI^-^ cells (early apoptotic cells) are in the fourth quadrant, and AV^+^ PI^+^ cells (late apoptotic cells) are in the second quadrant. ^###^*p* < 0.001 compared to the control group; ^∗^*p* < 0.05, ^∗∗^*p* < 0.01, and ^∗∗∗^*p* < 0.001 compared to the IH group.

Normal CMECs were negative for both AV and PI, early apoptotic CMECs were AV positive and PI negative, and late apoptotic and necrotic CMECs were positive for both AV and PI. Severe apoptosis was induced after CMECs were cultured in IH conditions for 46 h. Flow cytometry results are shown in **Figure 4**. When compared to the control group, the IH group of normal CMECs decreased significantly (*P* < 0.001), while in the VA-pro groups with a concentration of 0.25, 0.5, and 1 mg/ml, the number of apoptotic cells decreased (*P* < 0.001). These results suggested that VA-pro reduced the apoptosis of CMECs.

### VA-pro Regulating Effect on the Mitochondrial Membrane Potential (MMP) of CMECs Subjected to Ischemia-Hypoxia

**FIGURE 5:**
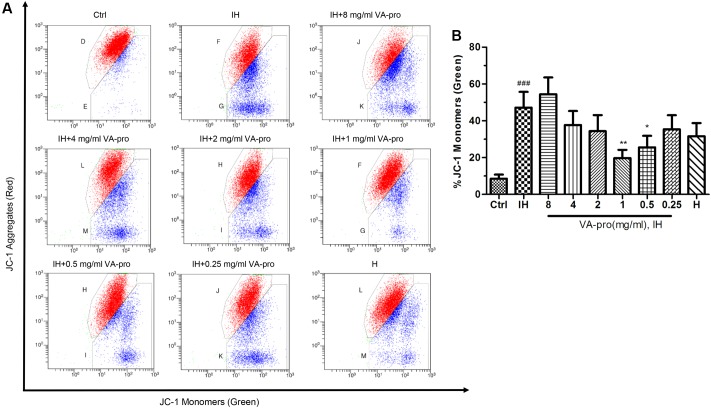
VA-pro kept the stability of CMECs MMP that were cultured in the IH conditions. MMP was assessed with a JC-1 kit **(A)**, histogram is the data analysis of the picture **(B)**. The horizontal axis of the flow cytometric dot plot is the blue fluorescence signal (JC-1 monomers) collected by the FL-1 channel; the vertical axis is the red fluorescent signal (JC-1 aggregates) collected by the FL-2 channel. ^###^*p* < 0.001 compared to the control group; ^∗^*p* < 0.05 and ^∗∗^*p* < 0.01 compared to the IH group.

The horizontal axis of the flow cytometric dot plot is the blue fluorescence signal (JC-1 monomers) and the vertical axis is the red fluorescent signal (JC-1 aggregates). **Figure 5** shows that there is a lower red fluorescent intensity and a higher blue fluorescent intensity in the IH group of CMECs (*P* < 0.001); this means that MMP decreased markedly when CMECs were subjected to IH, but MMP was increased when the cells were treated with VA-pro, especially at 0.5 and 1 mg/ml (*P* < 0.05). It indicated that VA-pro retained the stability of the MMP of CMECs that were cultured in the IH conditions.

### VA-pro Promoted the Migration of CMECs Subjected to Ischemia-Hypoxia

**FIGURE 6:**
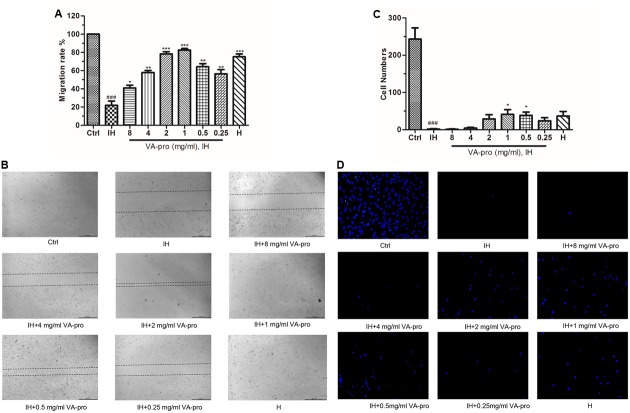
VA-pro promoted the migratory ability of CMECs subjected to IH. Scratch assay (**B**: magnification: ×50, scale bar: 500 μm) and transwell assay (**D**: cells were stained with DAPI, magnification: ×200, scale bar: 100 μm) were applied to the migratory ability of CMECs. For scratch assay, the control group migration rate was set at 100% **(A)**, for transwell assay, only 0.5 and 1 mg/ml dose groups showed good results **(C)**. ^###^*p* < 0.001 compared to the control group; ^∗^*p* < 0.05, ^∗∗^*p* < 0.01 and ^∗∗∗^*p* < 0.001 compared to the IH group.

For the scratch assay, as shown in **Figures 6A,B**, the control group migration rate was set at 100%, and quantitative analysis indicated that the migration rate of the IH group was decreased (*P* < 0.001). VA-pro treatment (0.25, 0.5, 1, and 2 mg/ml dose) reversed the migration rate as the wound areas of these groups were decreased compared to the IH group, while the 1 mg/ml and 2 mg/ml VA-pro groups had the best migratory ability (*P* < 0.001). This indicated that VA-pro promoted the migration of CMECs subjected to IH. Meanwhile, a better cell morphology was observed in the 1 mg/ml-VA-pro group under a microscope. For transwell assay, only 0.5 and 1 mg/ml dose groups showed good results (*P* < 0.05) (**Figures 6C,D**).

### VA-pro Maintained the Tube Formation Ability of CMECs Subjected to Ischemia-Hypoxia

**FIGURE 7:**
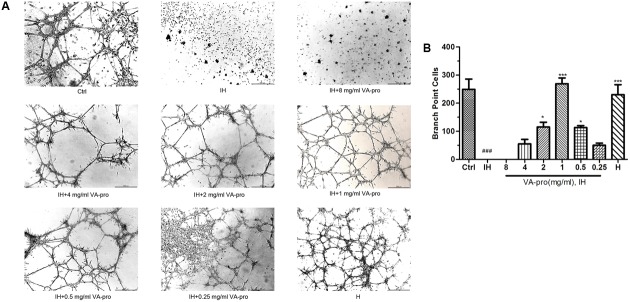
VA-pro maintained the tube formation ability of CMECs subjected to IH. **(A)** These images (magnification: ×50, scale bar: 500 μm) were analyzed using NIS Elements AR Analysis 4.10.00. **(B)** ^###^*p* < 0.001 compared to the control group; ^∗^*p* < 0.05 and ^∗∗∗^*p* < 0.001 compared to the IH group.

*In vitro*, endotheliocytes can spontaneously form a tubular capillary-like network in Matrigel culture. As shown in **Figure 7A** and quantified in **Figure 7B**, CMECs’ tubule formation ability was decreased when CMECs were subjected to IH (*P* < 0.001). VA-pro treatment (0.5, 1, and 2 mg/ml dose) potentially reversed the CMECs’ tubule formation ability that had been reduced by IH, especially at 1 mg/ml (*P* < 0.001), so VA-pro maintained the tube formation ability of CMECs.

### VA-pro Protected CMECs by Activating the PI3K/Akt Signaling Pathway

**FIGURE 8:**
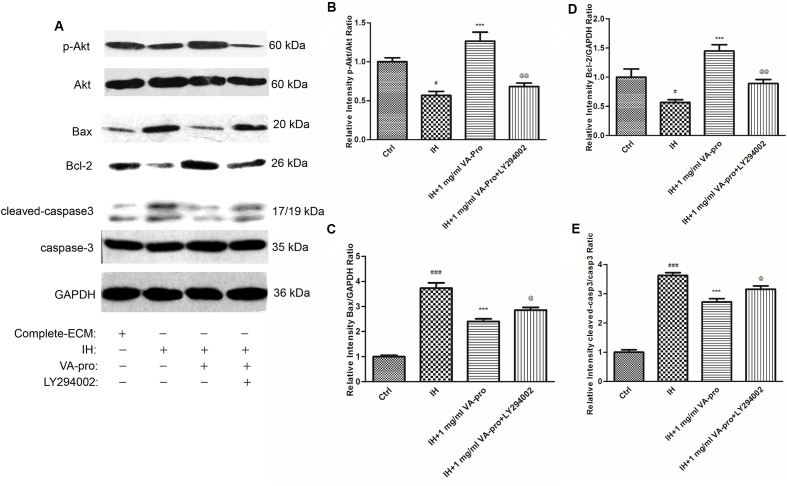
VA-pro protected IH-induced CMECs injury by activating the PI3K/Akt signaling pathway. Considering the results of **Figures 3**–**7**, we only retained the 1 mg/ml VA-pro group **(A)**. The IH treatment led to a decrease in the expression of p-Akt **(B)** and Bcl-2 **(D)**; in contrast, it increased the expression of Bax **(C)** and the cleaved caspase-3 **(E)**. Nonetheless, VA-pro reversed the cleaved caspase-3 and Bcl-2/Bax expression while upregulating the phosphorylation levels of Akt. In addition, the inhibitor of PI3K/Akt (LY294002) treatment suppressed the effects of VA-pro on Bax, Bcl-2, and cleaved caspase-3 expression, indicating that VA-pro protected IH-induced CMEC injury possibly through activating the PI3K/Akt signaling pathway. ^###^*p* < 0.001 compared to the control group; ^∗∗∗^ *p* < 0.001 compared to the IH group; ^@^*p* < 0.05 and ^@@^*p* < 0.01 compared to the “IH + 1 mg/ml VA-pro” group.

Considering the results of **Figures 3**–**7**, we only retained the 1 mg/ml VA-pro group. As shown in **Figure 8**, a lower expression of phosphorylated Akt (**Figures 8A,B**) and Bcl-2 (**Figures 8A,D**), and a higher expression of Bax (**Figures 8A,C**) and cleaved caspase-3 (**Figures 8A,E**) were observed in the IH group. VA-pro could reverse these changes. Further, the inhibitor of PI3K/Akt (LY294002) treatment suppressed the effects of VA-pro on Akt, Bax, Bcl-2, and cleaved caspase-3 expression, indicating that VA-pro protected the CMECs challenged with IH by activating the PI3K/Akt signaling pathway.

## Discussion

According to Chinese Pharmacopoeia, VA is a young antler of male sika deer (*Cervus nippon* Temminck) or male red deer (*Cervus elaphus*) with dense hair and it is the particular mammalian organ capable of repeated regeneration (Li et al., 2004; Price et al., 2005; Molnar et al., 2007), VA can grow rapidly within a few days to be a mature organ that has a powerful vascular system with the function of osteogenesis, hematopoiesis, and nerve, epidermal, and hair growth. Based on the Chinese medicine theory of cardionephric harmony, we believe that VA shows efficacy to treat heart diseases, and VA has already been employed to treat heart diseases in the clinic in China. Some animal experiments have shown that VA peptides have protective effects on acute ischemic myocardial injury (Chen et al., 2009) and cardiac functions of rats with heart failure following myocardial infarction (Shao et al., 2012). However, more studies on the effective components and the active mechanism of VA are needed.

Encouragingly, a series of studies suggested that proteins and peptides are the main substances of VA that promote cell proliferation (Zhou et al., 1999; Guan et al., 2006), and a variety of cell growth factors have been found in VA, such as epidermal growth factor (EGF) (Barling et al., 2005), nerve growth factor (NGF) (Pita-Thomas et al., 2010), fibroblast growth factor (FGF-2), and vascular endothelial growth factor (VEGF) (Clark et al., 2006; Lai et al., 2007). Our recent research showed that VA-pro can promote endothelial progenitor cell proliferation and migration via regulating the PI3K/Akt signaling pathway and Notch1 signaling pathway, but VA-pro had no effect on tube formation ability (Xiao et al., 2017). We wondered whether VA-pro may also have an effect on vascular endothelial cells or if it can be used to treat cardiovascular disease by influencing the CMECs function.

It is well documented that endothelial dysfunction is a crucial event in the development of cardiovascular diseases, including atherosclerosis, myocardial infarction, myocardial ischemia reperfusion, and hypertension. When vascular injury occurs, the partial regeneration of capillaries support the blood supply; this process mainly depends on vascular endothelial cell proliferation, migration, tube formation ability, and other functions. Although clinics commonly use revascularization treatments to open lesion vessels and save ischemic tissues or organs, they do not show good improvement in the function of tissues or organs. On the contrary, it brings ischemia-reperfusion injury problems. Currently, in clinical treatment, we put more emphasis on endothelial function and trying to protect endothelial cells from injury in the process of the disease. Some clinical drugs can regulate endothelial function and protect endothelial from injury, but there are few natural drugs that have a similar function.

Our results reported in this article reveal that CMECs’ proliferation, migration, and tube formation ability were seriously affected in the ischemia-hypoxia condition, with increasing apoptosis and the loss of basic function. The morphological structure of the cells also changed, cells shrunk, and cell connections disappeared. In addition, the apoptosis process is often accompanied by the destruction of the MMP, and it is widely considered to be the earliest events in the process of apoptosis. Our results showed that MMP decreased or disappeared when CMECs were subjected to ischemia-hypoxia. Interestingly, the use of VA-pro in the culture medium changed the above mentioned features and its effect on the MMP of CMECs coincided with the apoptotic results. Thus, the cells were able to resist the injuries caused by ischemia-hypoxia, and maintained good cell morphology and function.

PI3K is the main regulator of the Akt signaling pathway, and the PI3K/Akt signaling pathway participates in various cellular processes, such as cell survival, proliferation, apoptosis, and tube formation. A previous study showed that the activation of the PI3K/Akt signaling pathway also promotes CMEC migration, which is one of the critical events for angiogenesis (Cao et al., 2012). Other research has proven that the PI3K/Akt signaling pathway is the predominant mediator of ischemic postconditioning-induced cardioprotection (Zhu et al., 2006).

Caspase-3 is a crucial enzyme of the apoptotic pathway, and its activation is a central part of caspase-dependent apoptosis (Chan et al., 2010). Our results (**Figure 8**) showed a significant increase in caspase-3 activity in CMECs subjected to ischemia-hypoxia. These results indicated that IH-induced CMEC death may occur through a caspase-dependent apoptosis process. In addition, caspase-3 can cause DNA degradation, so its results were similar with the EdU Kit in this research. The Bcl-2 family can regulate the mitochondrial apoptotic pathway and determine whether mitochondria start the apoptosis program. Bcl-2 is an anti-apoptotic protein, while Bax is a pro-apoptotic protein, and they constitute a critical intracellular checkpoint for apoptosis. The balance between the two proteins is essential for cell survival against injuries caused by ischemia-hypoxia (Gustafsson and Gottlieb, 2007). As shown in our Western blot results, VA-pro could reverse the change in expression of Akt, caspase-3, Bcl-2, and Bax induced by ischemia-hypoxia. Furthermore, LY294002 is a PI3K inhibitor; these changes suggested that the PI3K/Akt signaling pathway may mediate the protective effect of VA-pro on CMECs.

**FIGURE 9:**
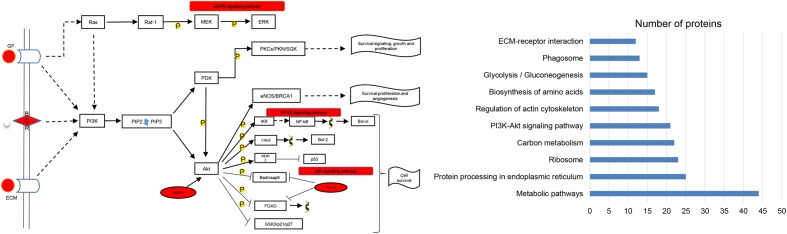
Signaling pathway analysis of VA-pro (KEGG database). The contents, marked in red, were the VA-pro involved in the signaling pathway. The bar graph shows the top 10 in the KEGG analysis results.

In addition to the cells experiment results, the protein signaling pathway analysis of VA-pro found that 21 proteins are directly related to the PI3K/Akt signaling pathway. The relevant information from the KEGG search is shown in **Figure 9**.

The data analysis of VA-pro in conjunction with the cell experiments reveal the mechanism behind the effects of VA-pro on CMECs subjected to ischemia-hypoxia. The composition of VA-pro is complex and the primary culture CMECs are sensitive and instable, thus, the dose-effect relationship was not obvious in the experiment, so we focused on the effect and probable dose range of VA-pro.

In summary, the results revealed that VA-pro has protective effects on the CMECs challenged with ischemia-hypoxia. The study provides a reference for the protection of CMECs, and it will be interesting and worthwhile to investigate the effect of VA-pro in CMECs subjected to ischemia-hypoxia. While although VA-pro has the potential therapeutic effect, but it is still a crude extract and its composition is complex. VA-pro needs to be separated and purified with deepening research and expanding experiments. So more research on the real active components and the concentration-response relationship is necessary.

## Ethics Statement

This study was carried out in accordance with the recommendations of “The Animal Care and Welfare Committee of China-Japan Friendship Hospital.” The protocol was approved by the “The Animal Care and Welfare Committee of China-Japan Friendship Hospital.”

## Author Contributions

XX carried out the experimental work and wrote the paper. SX and LH designed and supervised the experiments and revised the primary manuscript. MM, LL, and FY were responsible for the quality control and pharmaceutical analysis. JW and YL participated in the data analysis. ZW participated in the cell culture.

## Conflict of Interest Statement

The authors declare that the research was conducted in the absence of any commercial or financial relationships that could be construed as a potential conflict of interest.

## Footnotes

1 http://www.uniprot.org/

2 http://www.geneontology.org/

3 http://www.genome.jp/kegg/

## Abbreviations

CD31: Platelet endothelial cell adhesion molecule-1
CMECs: cardiac microvascular endothelial cells
GO: Gene Ontology
IH: ischemia hypoxia
KEGG: Kyoto Encyclopedia of Genes and Genomes
MMP: mitochondrial membrane potential
MW: proteins molecular weight
Nano HPLC-MS/MS: nanoliter liquid chromatography tandem mass spectrometry
pI: Protein isoelectric point
VA: velvet antler
VA-pro: velvet antler proteins
vWF: von Willebrand factor.
